# Supplementary material for: Racial disparities in diabetes care and outcomes for people with visual impairment: a descriptive analysis of the TriNetX research network
Source: BMC Public Health. 2025 Jul 19;25:2508. doi: 10.1186/s12889-025-23606-2 (PMC12275416; doi:10.1186/s12889-025-23606-2)
Supplement: Supplementary file 1 — Supplementary Material 1. [file 12889_2025_23606_MOESM1_ESM.docx]

Supplemental Materials

**Supplemental Table 1.** Visual disability-related ICD-10 codes used in this study

| **ICD-10 Codes** | **Diagnosis Category** | **Analysis Category** |
| --- | --- | --- |
| No H54.* Dx or One Eye Normal (H54.4*, H54.5*, H43.6*) | None | (-) VDRC |
| Unqualified or Unspecified Vision Loss (H54.3, H54.7) | Unqualified | (+) VDRC |
| Low Vision in Both Eyes or Better Eye (H54.1*, H54.2*) | Low Vision |  |
| Blindness in Both Eyes or Legal Blindness (H54.0*, H54.8) | Blindness |  |


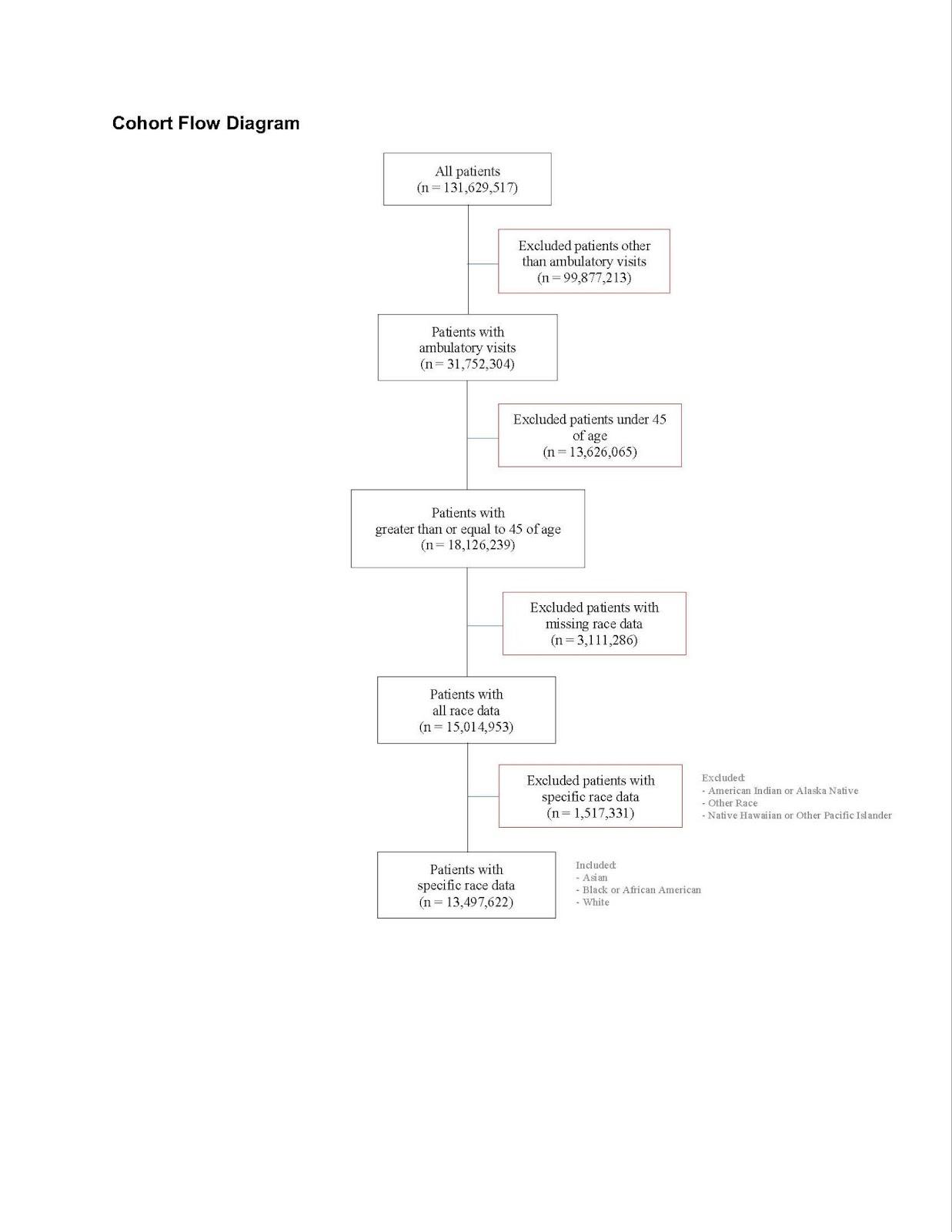

**Supplemental Figure 1. Study flow diagram.**

**Supplemental Table 2. Counts by outcome for each cohort before and after propensity score matching.**

| **VDRC** | **Race** | **Cohort** | **Patient Count** (Before Matching) | **Patient Count** (After Matching) |
| --- | --- | --- | --- | --- |
| No VDRC | AA or Black | 3+ visits | 126,227 | 3,732 |
|  | AA or Black | 1+ A1C | 87,179 | 2,464 |
|  | AA or Black | 1+ GFR | 107,860 | 3,095 |
|  | AA or Black | Comorbidity | 173,837 | 4,724 |
|  | White | 3+ visits | 584,002 | 18,320 |
|  | White | 1+ A1C | 399,568 | 9,830 |
|  | White | 1+ GFR | 436,476 | 11,393 |
|  | White | Comorbidity | 814,782 | 23,538 |
|  | Asian | 3+ visits | 53,293 | 477 |
|  | Asian | 1+ A1C | 39,943 | 315 |
|  | Asian | 1+ GFR | 46,300 | 365 |
|  | Asian | Comorbidity | 75,090 | 653 |
| VDRC Present | AA or Black | 3+ visits | 3,866 | 3,732 |
|  | AA or Black | 1+ A1C | 2,466 | 2,464 |
|  | AA or Black | 1+ GFR | 3,097 | 3,095 |
|  | AA or Black | Comorbidity | 4,724 | 4,724 |
|  | White | 3+ visits | 18,320 | 18,320 |
|  | White | 1+ A1C | 9,832 | 9,830 |
|  | White | 1+ GFR | 11,540 | 11,393 |
|  | White | Comorbidity | 23,538 | 23,538 |
|  | Asian | 3+ visits | 484 | 477 |
|  | Asian | 1+ A1C | 315 | 315 |
|  | Asian | 1+ GFR | 367 | 365 |
|  | Asian | Comorbidity | 653 | 653 |

**Supplemental DAG Information**

**Causal effect identification**

***Adjustment (total effect)***
Exposure: Disability (Visual Impairment)

Outcome: Uncontrolled Diabetes

Biasing paths are open.

Minimal sufficient adjustment sets for estimating the total effect of Disability (Visual Impairment) on Uncontrolled Diabetes:

- Comorbid Condition

***Adjustment (direct effect)***
Exposure: Disability (Visual Impairment)

Outcome: Uncontrolled Diabetes

Biasing paths are open.

Minimal sufficient adjustment sets for estimating the direct effect of Disability (Visual Impairment) on Uncontrolled Diabetes:

- Demographics, Diabetes, Disability Status[i], SDOH, Scheduled Medical Visit

**DAG code**

dag {

bb="0,0,1,1"

"A1C Lab " [pos="0.900,0.830"]

"Comorbid Condition Code" [pos="0.237,0.926"]

"Comorbid Condition Diagnosis" [pos="0.227,0.702"]

"Comorbid Condition" [pos="0.246,0.455"]

"Diabetes Code" [pos="0.398,0.928"]

"Diabetes Diagnosis" [pos="0.464,0.729"]

"Disability (Visual Impairment)" [exposure,pos="0.212,0.100"]

"Disability Status[i] Code" [pos="0.691,0.946"]

"Disability Status[i]" [pos="0.690,0.791"]

"Scheduled Medical Visit" [pos="0.672,0.461"]

"Uncontrolled Diabetes" [outcome,pos="0.865,0.405"]

A1C [pos="0.883,0.578"]

Demographics [pos="0.675,0.094"]

Diabetes [pos="0.449,0.141"]

SDOH [pos="0.831,0.145"]

Site [pos="0.351,0.558"]

"Comorbid Condition Diagnosis" -> "Comorbid Condition Code"

"Comorbid Condition" -> "Comorbid Condition Diagnosis"

"Comorbid Condition" -> "Disability (Visual Impairment)"

"Comorbid Condition" -> "Scheduled Medical Visit"

"Diabetes Diagnosis" -> "Diabetes Code"

"Disability (Visual Impairment)" -> "Disability Status[i]"

"Disability (Visual Impairment)" -> "Scheduled Medical Visit"

"Disability (Visual Impairment)" -> Diabetes

"Disability Status[i]" -> "Disability Status[i] Code"

"Disability Status[i]" -> "Uncontrolled Diabetes"

"Scheduled Medical Visit" -> "A1C Lab "

"Scheduled Medical Visit" -> "Disability Status[i]"

"Scheduled Medical Visit" -> "Uncontrolled Diabetes"

"Uncontrolled Diabetes" -> A1C

A1C -> "A1C Lab "

Demographics -> "Comorbid Condition"

Demographics -> "Uncontrolled Diabetes"

Demographics -> Diabetes

Diabetes -> "Diabetes Diagnosis"

Diabetes -> "Uncontrolled Diabetes"

SDOH -> "Comorbid Condition"

SDOH -> "Scheduled Medical Visit"

SDOH -> "Uncontrolled Diabetes"

SDOH -> Diabetes

SDOH -> Site

Site -> "A1C Lab "

Site -> "Comorbid Condition Code"

Site -> "Diabetes Code"

Site -> "Disability Status[i] Code"

Site -> "Disability Status[i]"

}
